# Dissociative symptoms with intravenous ketamine in treatment-resistant depression exploratory observational study

**DOI:** 10.1097/MD.0000000000026769

**Published:** 2021-07-23

**Authors:** Adam Włodarczyk, Wiesław J. Cubała, Maria Gałuszko-Węgielnik, Joanna Szarmach

**Affiliations:** Department of Psychiatry, Faculty of Medicine, Medical University of Gdańsk, Poland.

**Keywords:** dissociation, ketamine, psychosis, safety, tolerability, treatment-resistant depression

## Abstract

There is evidence for ketamine use in treatment-resistant depression (TRD). Several safety and tolerability concerns arise regarding adverse drug reactions and specific subpopulations. This paper aims to investigate the relationship between dissociative and psychometric measures in course of intravenous ketamine treatment in TRD inpatients with major depressive disorder and bipolar disorder.

This study result represents safety data in a population of 49 inpatients with major depressive disorder and bipolar disorder subjects receiving eight 0.5 mg/kg of ketamine intravenous infusions, with a duration of 40 min each, as an add-on treatment to standard-of-care pharmacotherapy, registered in the naturalistic observational protocol of the tertiary reference unit for mood disorders (NCT04226963). The safety psychometrics assessed dissociation and psychomimetic symptomatology with the Clinician-Administered Dissociative States Scale (CADSS) the Brief Psychiatric Rating Scale (BPRS).

The significant differences in CADSS scores between measurements in course of the treatment were observed (*P* = .003). No significant differences between BPRS measurements were made after infusions. In each case, both BPRS and CADSS values dropped to the “absent” level within 1 hour from the infusion. Neither CADSS nor BPRS scores were associated with the treatment outcome.

The study demonstrates a good safety profile of intravenous ketamine as an add-on intervention to current psychotropic medication in TRD. The abatement of dissociation was observed in time with no sequelae nor harm. The study provides no support for the association between dissociation and treatment outcome.

This study may be underpowered due to the small sample size. The protocol was defined as a study on acute depressive symptomatology without blinding.

## 1 Introduction

Recent developments in rapid-acting antidepressant use in treatment-resistant depression (TRD) provide robust evidence for ketamine use in major depressive disorder (MDD) and bipolar disorder (BP) providing an option for rapid remission of symptoms with several concerns arising with regards to safety and tolerability of the drug.^[1–4]^

One of the major issues is the risk of adverse events associated with dissociative symptomatology.^[5]^ There is some evidence for dissociative symptoms as the predictor of response in TRD (both TRD-MDD and TRD-BP), however, it is limited to very few papers.^[2]^ There is scarce data on dissociative symptomatology in ketamine-treated depressive patients.^[4]^ With some indication of long-term symptoms, time course characteristics originating from esketamine studies^[6–8]^ demonstrating the abatement of dissociative symptoms in time. It is hypothesized that dissociation is associated with the treatment outcome, while a body of evidence does not support this finding. Although the pathophysiology of mood disorders is pleomorphic, some diseases can contribute to dissociation.^[9,10,11]^

Dissociative symptoms cause a wide spectrum of phenomena, however, per methodological guidance the Clinician-Administered Dissociative States Scale (CADSS) and Brief Psychiatric Rating Scale (BPRS) with positive symptoms subscale are used. That may represent the overall intensity of the dissociative symptomatology.^[1–8,12]^

The aim of this paper is to investigate the relationship between dissociative measures and psychometric outcomes in course of intravenous ketamine treatment in treatment-refractory inpatients with MDD and BP.

## 2 Methods and population

The study population selection has been described in detail elsewhere.^[13,14,15]^ As being stated in the cited papers, the study participants were included in a naturalistic observational safety and efficacy registry protocol for ketamine infusions in TRD. Inpatients diagnosed with a depressive episode in the course of major depression (35 subjects) or BP (14 subjects), both conditions without psychotic features, were included. Subjects were examined with Mini-International Neuropsychiatric Interview by clinicians to verify the diagnosis using Diagnostic and Statistical Manual of Mental Disorders criteria. All participants exhibited treatment resistance for the current episode, defined as an inadequate response to 2 or more antidepressants (assessed by Massachusetts General Hospital Antidepressant Treatment Response Questionnaire), with some patients fulfill the treatment-refractory criteria, which refers to 3 or more failures including electroconvulsive therapy^[16]^ and the “difficult to treat” depression that “*continues to cause significant burden despite usual treatment efforts*”^[17]^ in course of treatment of that particular episode in unipolar depression. As for bipolar depression, we defined TRD as a clinically unsatisfactory response following at least 2 trials of dissimilar medicinal treatments in presumably adequate doses and durations, within a specific phase of bipolar illness.^[18]^ On inclusion, the major depressive episode severity was 28 points in Montgomery–Åsberg Depression Rating Scale (MADRS) (SD ± 7.52). The study followed the rule of single-patient and single-rater. During the screening patients were rated by the clinician using MADRS, Young Mania Rating Scale, Columbia–Suicide Severity Rating Scale, The CADSS), BPRS. The CADSS was chosen as it is the most widely used instrument to assess the acute psychoactive effects of ketamine administration in past trials for mood disorders^[19]^ and the BPRS with positive symptoms subscale, the 4-item positive symptom subscale from the BPRS as a safety assessment.^[8]^

Only medically stable, able to communicate and provide consent, adult inpatients aged 18–65 were enrolled to study. Some patients significantly affected by somatic illness continued current medication during ketamine treatment. The exclusion criteria comprised of a history of uncontrolled medical conditions, a history of adverse reaction to ketamine, psychoactive substance abuse, pregnancy, or breastfeeding. All subjects gave written informed consent to participate in the study. The study was carried out in accordance with the latest version of the Declaration of Helsinki. For each participant, written consent was obtained after the procedures had been fully explained. The study protocols were approved by the Independent Bioethics Committee for Scientific Research at Medical University of Gdańsk, Poland: NKBBN/172/2017; 172-674/2019. The study population comprises MDD and BP subjects treated with ketamine registered in the naturalistic observational protocol of the tertiary reference unit for mood disorders (NCT04226963); https://clinicaltrials.gov/ct2/show/NCT04226963.

### 2.1 Study design: ketamine infusions

The study followed an observational design with all patients continuing baseline psychotropic standard-of-care, as well as treatment of chronic somatic diseases during ketamine infusions. The study therapeutic intervention was based on the administration of 8 ketamine infusions over 4 weeks. Ketamine was dosed at 0.5 mg/kg based on the patient's actual body weight and infused intravenously over 40 min for all of the infusions.

Safety monitoring was performed by the attending psychiatrist before, during, and post-infusion every 15 minutes up to an hour and a half post-infusion. It included periodic assessment of vital signs (heart rate, body temperature, respiratory rate, blood pressure, and oxygen saturation) and mental status examination, including assessment of BPRS and CADSS for the presence of respectively psychotic and dissociative symptoms, which were measured before, 40 minutes and 1 hour after the infusion. Any other significant adverse effect (eg, nausea) was also monitored. Psychometric evaluation with Young Mania Rating Scale and MADRS was done prior to the 1st, 3rd, 5th, and 7th infusion and during the follow-up (1 week after the last infusion). The electrocardiogram was carried out before every second infusion and 1 week after the last ketamine infusion. All clinicians were professional psychiatrists, were also familiar with behavioral management of patients with marked mental status changes, and were prepared to take care of any emergency behavioral situations. Furthermore, an on-site clinician was available and evaluated the patient for potential behavioral risks, including suicidal ideation, after the end of each session.

A subject was defined as a responder at a given time point if the percent improvement from baseline in MADRS total score was at least 50%. A subject was defined as being in remission at a given time point if the MADRS total score was ≤10 points.^[20]^ The groups (remitters, responders, and non-remitters) were determined at the end of the study, during follow-up, when the final MADRS evaluation took place.

### 2.2 Statistical analysis

The analyzes were conducted using statistical software the IBM SPSS Statistics 25.0. To determine the differences between responders, remitters, and non-responders for sociodemographic variables and the occurrence of diseases and treatment, frequency analyzes were carried out with Fisher exact test. Analysis for quantitative variables was carried out by the Kruskal–Wallis test.

To compare the 2 groups in terms of ordinal or quantitative variables, the Mann–Whitney *U* test analysis was performed. When there were more groups, the analysis was carried out by the Kruskal–Wallis *H* test. To determine the relationship between 2 quantitative variables, Spearman rank correlation analysis was performed.

To determine the differences between measurements, mixed models analysis was used.

The medium-term rate of change of the analyzed variables was calculated using chain indexes – the harmonic average of all chain indexes was calculated. Based on the medium-term rate of change, the rate of change was calculated for a given variable and the relationships between the dynamics of change between variables were determined. α = 0.05 was adopted as the level of significance for this analysis.

Due to the small sample size, non-parametric tests were used, and in acuities among groups analyzed were used for the analysis for discrete and continuous variables.

## 3 Results

The clinical and demographic characteristic of the study group is presented in Table 1.

**Table 1:** Demographic and clinical variables.

		N	Responder	Remitter	Non-responder	*P*	*V*
Male, sex	(%)	21 (42.9)	6 (66.7)	2 (25.0)	13 (40.6)	.229	0.26
Age, in years		50.02 (13.83)	53.11 (7.06)	42.88 (15.78)	50.94 (14.51)	.336^1^	0.00
BMI		27.92 (5.67)	28.00 (4.64)	26.50 (4.72)	28.25 (6.21)	.613^1^	0.02
Ketamine Treatment for:							
	MDD	35 (71.4)	8 (88.9)	5 (62.5)	22 (68.8)	.475	0.19
	BP	14 (28.6)	2 (11.1)	5 (37.5)	7 (31.2)	.485	0.18
							
							
Comorbidities						.104	0.31
	1	21 (42.9)	6 (66.7)	2 (25.0)	13 (40.6)		
	2	10 (20.4)	2 (22.2)	1 (12.5)	7 (21.9)		
	3	4 (8.2)	1 (11.1)	2 (25.0)	1 (3.1)		
	Hypertension	16 (32.7)	6 (66.7)	3 (37.5)	7 (21.9)	.037	0.37
	BP	4 (8.2)	1 (11.1)	2 (25.0)	1 (3.1)	.052	0.66
	MDD	12 (24.5)	5 (55.6)	1 (12.5)	6 (18.8)	.177	0.33
	Diabetes mellitus	3 (6.1)	1 (11.1)	2 (25.0)	0 (0)	.021	0.39
	Hyperlipidemia	9 (18.4)	3 (33.3)	1 (12.5)	5 (15.6)	.545	0.19
	Post-stroke	3 (6.1)	1 (11.1)	0 (0)	2 (6.3)	.731	0.14
	Post-myocardial infarct	0 (0)	0 (0)	0 (0)	0 (0)	-	-
	Epilepsy	6 (12.2)	0 (0)	3 (37.5)	3 (9.4)	.060	0.36
	Other	16 (32.7)	2 (22.2)	1 (12.5)	13 (40.6)	.330	0.24
Coexisting treatment							
	TCA	8 (16.3)	1 (11.1)	1 (13.5)	6 (18.8)	1.000	0.09
	Clomipramine	4 (8.2)	0 (0)	1 (12.5)	3 (9.4)	.789	0.15
	Amitriptiline	4 (8.2)	1 (11.1)	0 (0)	3 (9.4)	1.000	0.13
	SSRI total	23 (46.9)	5 (55.6)	2 (25.0)	16 (50.0)	.413	0.20
	Fluvoxamine	1 (2.0)	0 (0)	0 (0)	1 (3.1)	1.000	0.11
	Paroxetine	5 (10.2)	1 (11.1)	0 (0)	4 (12.5)	.813	0.15
	Fluoxetine	8 (16.3)	2 (22.2)	0 (0)	6 (18.8)	.534	0.20
	Sertraline	3 (6.1)	1 (11.1)	0 (0)	2 (6.3)	.731	0.14
	Citalopram	4 (8.2)	0 (0)	2 (25.0)	2 (6.3)	.179	0.29
	Escitalopram	2 (4.1)	1 (11.1)	0 (0)	1 (3.1)	.578	0.18
	SNRI total	11 (22.4)	2 (22.2)	2 (25.0)	7 (21.9)	1.000	0.03
	Venlafaxeine	8 (16.3)	1 (11.1)	1 (12.5)	6 (18.8)	1.000	0.10
	Duloxetine	3 (6.1)	1 (11.1)	1 (12.5)	1 (3.1)	.273	0.17
	Other					.749	0.14
	1	15 (30.6)	4 (44.4)	2 (25.0)	9 (28.1)		
	2	3 (6.1)	0 (0)	1 (12.5)	2 (6.3)		
	Mirtazapine	9 (18.4)	2 (22.2)	1 (12.5)	6 (18.8)	1.000	0.08
	Mianserine	3 (6.1)	1 (11.1)	0 (0)	2 (6.3)	.731	0.14
	Trazodone	4 (8.2)	1 (11.1)	1 (12.5)	2 (6.3)	.432	0.10
	Bupropione	3 (6.1)	0 (0)	1 (12.5)	2 (6.3)	.488	0.15
	Vortioxetine	2 (4.1)	0 (0)	1 (12.5)	1 (3.1)	.333	0.20
	Antipsychotic medication					.806	0.15
	1	12 (24.5)	2 (22.2)	1 (12.5)	9 (28.1)		
	2	5 (10.2)	0 (0)	1 (12.5)	4 (12.5)		
	Aripiprazole	6 (12.2)	0 (0)	1 (12.5)	5 (15.6)	.685	0.18
	Quetiapine	10 (20.4)	1 (11.1)	1 (12.5)	8 (25.0)	.668	0.16
	Olanzpiane	5 (10.2)	1 (11.1)	1 (12.5)	3 (9.4)	1.000	0.04
	Risperidone	1 (2.0)	0 (0)	0 (0)	1 (3.1)	1.000	0.11
	Mood stabilizers					.348	0.29
	1	15 (30.6)	2 (22.2)	4 (50.0)	9 (28.1)		
	2	6 (12.2)	1 (11.1)	0 (0)	5 (15.6)		
	3	1 (2.0)	0 (0)	1 (12.5)	0 (0)		
	lithium	5 (10.2)	0 (0)	1 (12,5)	4 (12.5)	.643	0.16
	valproate	9 (18.4)	2 (22.2)	3 (37.5)	4 (12.5)	.160	0.24
	lamotrigine	7 (14.3)	1 (11.1)	1 (12.5)	5 (15.6)	1.000	0.05

Mood stabilizers – based on Young (2004).^[32]^ BMI = body mass index, BP = bipolar disorder, MDD = major depressive disorder, SNRI = selective serotonin-noradrenaline reuptake inhibitors, SSRI = selective serotonin reuptake inhibitors, TCA = other than mentioned tricyclic antidepressants.

CADSS changes during and after infusion are shown in Table 2.

**Table 2:** The interaction effects of ketamine infusions on CADSS and BPRS.

	CADSS before infusion		CADSS after 40 minutes from infusion				
	*M* (SD)	Me (IQR)	*M* (SD)	Me (IQR)	*Z*	*P*	*r*
Infusion 1	0.18 (0.49)	0 (0)	13.63 (9.14)	14 (13)	−5.97	<.001	−0.85
Infusion 2	0.35 (1.48)	0 (0)	14.90 (11.92)	13 (15.5)	−5.97	<.001	−0.85
Infusion 3	0.55 (2.41)	0 (0)	11.43 (13.14)	8 (12)	−5.42	<.001	−0.77
Infusion 4	0.27 (1.30)	0 (0)	11.94 (11.58)	10 (12.5)	−5.72	<.001	−0.82
Infusion 5	0.22 (0.77)	0 (0)	10.63 (8.04)	9 (10.5)	−5.65	<.001	−0.81
Infusion 6	0.36 (1.58)	0 (0)	10.15 (8.80)	9,5 (13.5)	−4.70	<.001	−0.82
Infusion 7	0.22 (1.16)	0 (0)	9.53 (10.27)	6 (15)	−5.31	<0.001	−0.76
Infusion 8	0.24 (1.16)	0 (0)	8.16 (9.04)	5 (10)	−5.38	<.001	−0.77
Pre-post	0.45 (1.72)	0 (0)	0.90 (3.16)	0 (0)	−0.86	.389	−0.12

	BPRS before infusion		BPRS after 40 minutes from infusion				
	*M* (SD)	Me (IQR)	*M* (SD)	Me (IQR)	*Z*	*P*	*r*
Infusion 1	0.18 (0.67)	0 (0)	0.82 (1.50)	0 (1)	−3.17	.002	−0.45
Infusion 2	0.33 (1.07)	0 (0)	0.82 (1.41)	0 (1)	−3.09	.002	−0.44
Infusion 3	0.04 (0.20)	0 (0)	0.67 (1.95)	0 (1)	−3.33	.001	−0.48
Infusion 4	0.06 (0.32)	0 (0)	0.53 (0.87)	0 (1)	−3.75	<.001	−0.54
Infusion 5	0.06 (0.24)	0 (0)	0.59 (0.96)	0 (1)	−3.52	<.001	−0.50
Infusion 6	0.15 (0.88)	0 (0)	0.63 (1.10)	0 (1)	−3.94	<.001	−0.69
Infusion 7	0.02 (0.16)	0 (0)	0.25 (0.65)	0 (0)	−2.27	.023	−0.32
Infusion 8	0.16 (0.91)	0 (0)	0.39 (0.98)	0 (0.5)	−3.05	.002	−0.44
Pre-post	0.24 (0.95)	0 (0)	0.31 (1.10)	0 (0)	−0.97	.334	−0.14

BPRS = Brief Psychiatric Rating Scale, CADSS = Clinician-Administeredered Dissociative States Scale.

**Figure 1:**
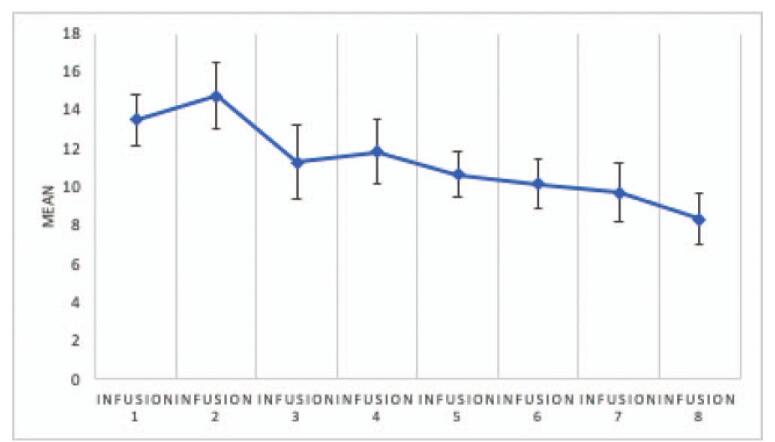
Means and standard errors for CADSS values measured after subsequent infusions (data apply to all subjects). *Infusion* – an intravenous infusion of ketamine. Ketamine was dosed at 0.5 mg/kg based on the patient's actual body weight and infused intravenously over 40 min for all of the infusions. CADSS = Clinician-Administeredered Dissociative States Scale.

**Figure 2:**
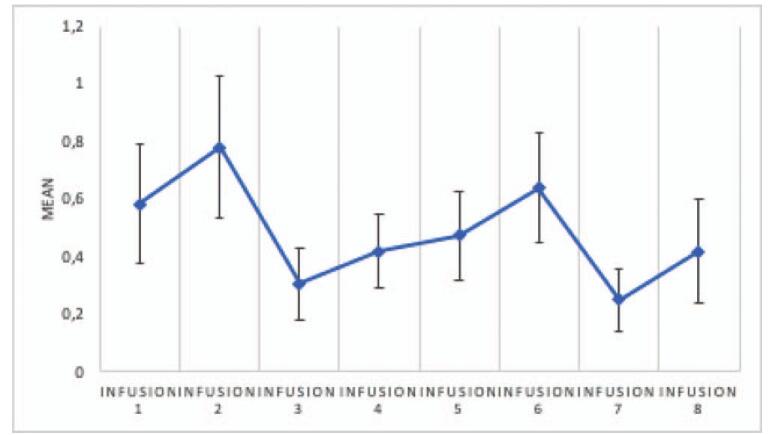
The CADSS scores over time. *Infusion* – an intravenous infusion of ketamine. Ketamine was dosed at 0.5 mg/kg based on the patient's actual body weight and infused intravenously over 40 min for all of the infusions. CADSS = Clinician-Administeredered Dissociative States Scale.

The analysis showed significant differences in CADSS scores between measurements, *F* (4.466) = 3.85; *P* = .003; and η2p = .08. The analysis showed that the only significant difference between measurements occurred between measurement for infusion 2 and infusion 8 (*P* = .02). Figure 1 illustrates the mean and standard errors for CADSS measurements. All CADSS values declined to an “absent” level within 1 hour after ketamine infusion in each patient at all times. The CADSS scores over time are presented in Figure 2.

The exploratory results of the analyzes are presented in Table 2.

BPRS changes during and after infusion are shown in Table 2.

**Figure 3:**
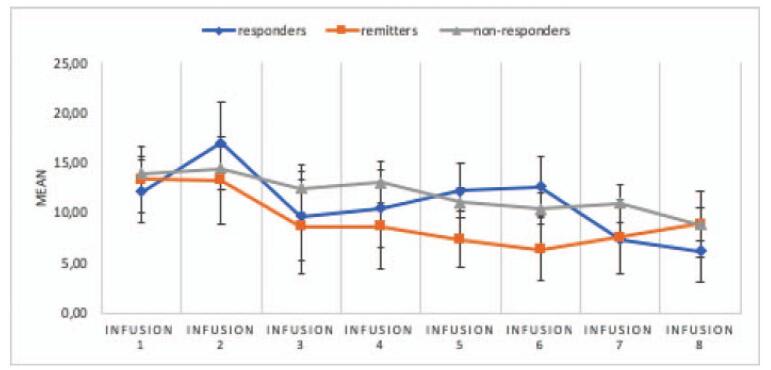
Means and standard errors for BPRS scores before and after the consecutive infusions (data apply to all subjects). *Infusion* – an intravenous infusion of ketamine. Ketamine was dosed at 0.5 mg/kg based on the patient's actual body weight and infused intravenously over 40 min for all of the infusions. BPRS = Brief Psychiatric Rating Scale.

The analysis showed no significant differences between BPRS measurements made after infusions, *F* (3.281) = 1.91; *P* = .12; and η2p = .05. Figure 3 illustrates the means and standard errors for BPRS measurements. All BPRS values declined to “absent” level within 1 hour after ketamine infusion in each patient every time.

### 3.1 Change over time in CADSS and MADRS scores

Post-hoc analysis of the results showed the presence of the main effect of CADSS, *F* (4.328) = 3.22; *P* = .01; and η2p = .07. The interaction effect of the group and the CADSS result proved to be insignificant, *F* (8.656) = .61; *P* = .78; and η2p = .03, which means that changes in CADSS time are independent of a group membership.

Post-hoc analysis of the results showed that the measurement made before and after the infusions did not differ significantly ().

**Figure 4:**
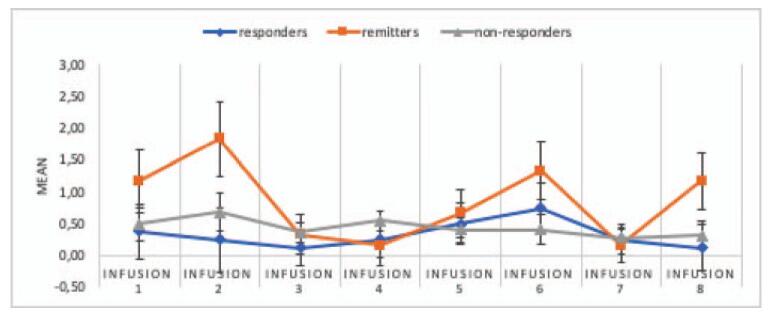
Means and standard errors for CADSS scores before and after the consecutive infusions in responders, remitters, and non-responders (data apply to all subjects). *Infusion* – an intravenous infusion of ketamine. Ketamine was dosed at 0.5 mg/kg based on the patient's actual body weight and infused intravenously over 40 min for all of the infusions. CADSS = Clinician-Administeredered Dissociative States Scale.

### 3.2 Change over time in BPRS and MADRS

**Figure 5:**
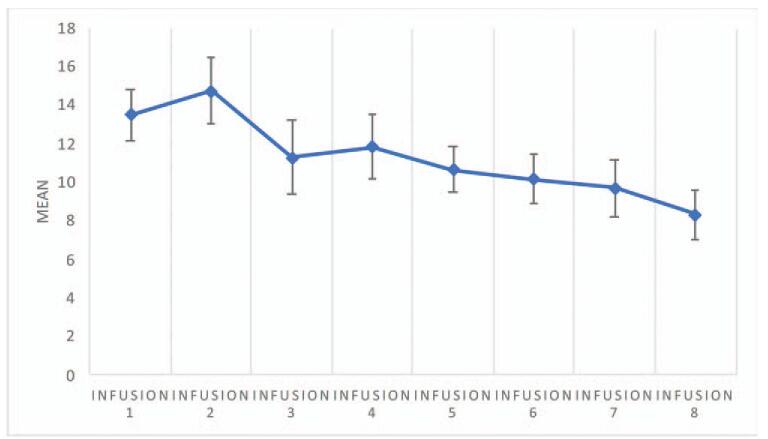
Means and standard errors for BPRS scores before and after the consecutive infusions in responders, remitters, and non-responders (data apply to all subjects). *Infusion* – an intravenous infusion of ketamine. Ketamine was dosed at 0.5 mg/kg based on the patient's actual body weight and infused intravenously over 40 min for all of the infusions. BPRS = Brief Psychiatric Rating Scale.

Post-hoc analysis of the results showed the occurrence of the main BPRS effect considering the division into groups, *F* (3.669) = 3.17; *P* = .02; and η2p = .09. The interaction effect of the group and the BPRS result was insignificant, *F* (7.333) = 1.7; *P* = .11; and η2p = .09. Post-hoc analysis of the results showed that the BPRS value after the 6th infusion is slightly higher than after the 8th infusion (*P* = .047). Figure 5 illustrates the means with standard errors for measurements.

## 4 Discussion

The research demonstrates good safety and tolerability profile of central nervous system (CNS) adverse drug reactions with ketamine as an add-on treatment to current psychotropic medication in TRD. The abatement of dissociation was demonstrated at the time of the treatment with no sequelae. The study provides no support for the association between CADSS score and treatment outcome with MADRS. Our study does not show the same efficacy as the esketamine nasal spray studies, however, this study includes the TRD population in a broad definition spectrum.

Our analysis showed significant differences for CADSS values before and after infusion in 1 measurement. CADSS values after infusion were significantly higher than before infusion for each of the 8 measurements. The effect strength for the differences was large. However, the difference between CADSS and BPRS measurements before and after each infusion turned out to be insignificant (pre- and post-measurements) (Table 2). The analysis showed significant differences for BPRS values before and after the infusion in 1 measurement. BPRS values after infusion were significantly higher than before infusion for each of the 8 measurements. The effect strength for the differences was large.

The methodological strength of our study was to strengthen the point that the tolerability and general safety of the administration of the drug and that result being in support with some previous ketamine studies mentioned above.

However, the study may be underpowered with regard to the small sample size. The research was performed as a single-site study, there was no treatment blinding, during this observational protocol study. The observations apply to treatment-resistant patients and include both, unipolar and bipolar depressed patients. Some concerns remain in terms of establishing an effective protocol to maintain the clinical antidepressant effect of ketamine seen with acute administration while managing long-term safety. Also, was that no CADSS assessment was obtained only once post-dose (30 minutes post-dose) without a few measurements time points, so we could not establish the precise time course of either the peak dissociative symptoms or of their resolution (thus, relying on the esketamine/ketamine literature, we expect that these side effects would have resolved by the 2-hour time point, but the data acquired here do not allow that interval. The findings provide support for further consideration of ketamine-related CNS symptomatology into the relevance of these stated in treatment outcomes. With no long-term psychotomimetic side-effects reported it is important to replicate the finding in a larger sample in the long-term safety study design to demonstrate no sequelae.

Our study is in line with esketamine trials^[6–8]^ as it was shown to produce no harm with esketamine treatment and all of the patients experienced any persistent dissociative or psychotic symptoms during the follow-up visit. In the above-cited phase III trials the dissociation was reported by 12.5% to 27.6% of patients, typically mild (<4% reported as severe) and transient: it reached its peak at 40 minutes, was typically resolved within 90 minutes, and tendency to reduce in intensity with repeated dosing was reported.^[6–8]^ Initially postulated effect by mentioned studies^[21–23]^ on the impact of dissociative/psychotic symptoms was not observed, being in line with esketamine-nasal spray trials.

Ketamine-induced central nervous symptomatology including dissociation and psychosis are believed to be associated with *N*-methyl-d-aspartate receptor blockade and surfeit ketamine activity.^[23]^ There is significant interest in whether the intensity of the acute ketamine experience is correlated with the ultimate antidepressant response.^[21]^ A performed literature review of the relationship between the antidepressant and CNS tolerability (dissociative or hallucinogenic effects) of ketamine found that only a few studies supported a weak to moderate degree of association. In agreement with previous reports^[1,21–27]^ ketamine was found to elevate CNS symptoms, described by the CADSS and BPRS assessed dissociative/psychotic effects.

Three CADSS studies found significant correlations, of which 1 with regards to CADSS depersonalization subscale^[28]^ and 1 BPRS study demonstrated correlations.^[21–23,28]^ However even larger number of studies revealed no significant relationship between CADSS and/or BPRS results and depression outcomes.^[1,8,19,24–27]^ Although, both CADSS and BPRS values after infusion were significantly higher than before infusion for each of 8 measurements, the difference for CADSS and BPRS measurements before and after each infusion turned out to be insignificant (pre- and post-treatment measurements). Although the intensity of CADSS scoring was higher than in esketamine nasal spray ^[8]^ the abatement with time as well as a self-limiting course with no sequelae was demonstrated in our study.

The presented by our team study provides no support for the intensity of dissociative symptomatology as being associated with an anti-depressive treatment effect of ketamine in TRD. We investigated the safety and tolerability of dissociative and/or psychotic symptoms as induced by the anti-depressive drug ketamine and also their possible association with better response with depression reduction. Ketamine is to be preferred in mood disorders treatment due to its safety and efficacy, which seems to duplicate multiple populations, including its use in incidental pain, wound dressing without any hemodynamic changes or sedation.^[29,30]^ This safety report contributes to the current literature on ketamine safety in mood disorders, in particular, to the TRD-BP population with limited data available to date. In addition, few studies are reporting on dissociation and the use of psychotropic drugs other than selective serotonin reuptake inhibitors/selective serotonin-noradrenaline reuptake inhibitors.^[31]^ Although speculative, the dissociation may be related to the psychotropic medication or the disease, instead of ketamine or its enantiomers use.

The amount of nonresponders is unexpectedly high, which may be accounted for the starting group with TRD and also patients with treatment-refractory symptomatology and also with coexisting somatic comorbidities. Thus, the patients varied with regards to both the diagnosis as well as the stage of the disease.

The study results demonstrate good safety and tolerability profile of CNS adverse drug reactions with short-term treatment with intravenous ketamine as an add-on intervention to current standard-of-care psychotropic medication in TRD. The abatement of dissociation was observed at the time of the treatment with no sequelae nor harm. The study provides no support for the association between dissociation and treatment outcome.

## Author contributions

**Conceptualization:** Adam Włodarczyk, Wieslaw Jerzy Cubała, Maria Gałuszko-Węgielnik.

**Formal analysis:** Adam Włodarczyk, Wieslaw Jerzy Cubała, Joanna Szarmach.

**Investigation:** Adam Włodarczyk, Maria Gałuszko-Węgielnik.

**Methodology:** Wiesław J. Cubała, Adam Włodarczyk, Maria Gałuszko-Węgielnik.

**Supervision:** Wieslaw Jerzy Cubała.

**Validation:** Wieslaw Jerzy Cubała.

**Writing – original draft:** Adam Włodarczyk.

**Writing – review & editing:** Joanna Szarmach, Adam Włodarczyk, Wieslaw Jerzy Cubała.
